# Does the Timing of Pre-Operative Medical Evaluation Influence Perioperative Total Hip Arthroplasty Outcomes?

**DOI:** 10.2174/1874325001711010195

**Published:** 2017-03-22

**Authors:** Roy H. Lan, Atul F. Kamath

**Affiliations:** 1 College of Arts and Sciences, Wharton School, University of Pennsylvania, Philadelphia, Pennsylvania, USA; 2 Department of Orthopaedic Surgery, Hospital of the University of Pennsylvania, Philadelphia, Pennsylvania, USA

**Keywords:** ICU admission frequency, Total hip arthroplasty, Length of stay in hospital, Major complications, Pre-operative evaluation, Total joint arthroplasty

## Abstract

**Background:**

Medical evaluation pre-operatively is an important component of risk stratification and potential risk optimization. However, the effect of timing prior to surgical intervention is not well-understood. We hypothesized that total hip arthroplasty (THA) patients seen in pre-operative evaluation closer to the date of surgery would experience better perioperative outcomes.

**Methods:**

We retrospectively reviewed 167 elective THA patients to study the relationship between the number of days between pre-operative evaluation (range, 0-80 days) and surgical intervention. Patients’ demographics, length of stay (LOS), ICU admission frequency, and rate of major complications were recorded.

**Results:**

When pre-operative evaluation carried out 4 days or less before the procedure date, there was a significant reduction in LOS (3.91 vs. 4.49; p=0.03). When pre-operative evaluation carried out 11 days or less prior to the procedure date, there was a four-fold decrease in rate of intensive care admission (p=0.04). Furthermore, the major complication rate also significantly reduced (p<0.05). However, when pre-operative evaluation took place 30 days or less before the procedure date compared to more than 30 days prior, there were no significant changes in the outcomes.

**Conclusion:**

From this study, pre-operative medical evaluation closer to the procedure date was correlated with improved selected peri-operative outcomes. However, further study on larger patient groups must be done to confirm this finding. More study is needed to define the effect on rare events like infection, and to analyze the subsets of THA patients with modifiable risk factors that may be time-dependent and need further time to optimization.

## INTRODUCTION

The volume of total joint arthroplasty procedures is increasing within the United States, as both the supply and demand have steadily climbed [1-3]. In particular, total hip arthroplasty (THA) procedures are becoming a more common orthopedic procedure [4, 5]. With this rise in procedure volume, it is important to understand the mechanisms by which procedure safety and quality can be improved [6]. Pre-operative medical evaluations are a crucial part of delivering robust, quality driven care for patients both within orthopedics and other fields of surgery [7-9]. The perioperative optimization of patients undergoing THA and revision procedures proves no exception [10]. While it is intuitive that conducting a thorough, robust pre-operative evaluation is critical to improving surgical outcomes, it is not as clear as to when pre-operative evaluations are most effective. As efficiency and throughput measures increase in importance in orthopedics, the ability to match pre-operative evaluation with the timing of surgery may prove important.

There are a number of studies within the literature that have examined best practices of pre-operative evaluations for a variety of medical procedures, but very few have examined when to conduct these examinations pre-operatively [11-14]. In fact, there are significant gaps within the literature regarding the timing of pre-operative evaluations, especially within orthopedics. There are no studies, to our knowledge on the total joint literature, that specifically examine the optimal timing of pre-operative evaluations as related to post-operative outcomes measures [15].

This study aims to determine whether the timing of pre-operative evaluations for patients undergoing THA affects perioperative health outcomes. More specifically, this study examines the relationship between the timing of pre-operative evaluations and three quality measures: length of stay (LOS) in hospital, need for higher acuity of care in-hospital (ICU admission frequency), and the frequency of major complications post-operatively.

## MATERIALS AND METHODS

This study included 175 patients who underwent either a THA or revision THA procedure from November 21, 2011 to June 11, 2012. The data set was obtained directly from the institutional data requisition department under a Strategic Decision Support (SDS) facility. Complete data was available for 167 patients, which comprised the final cohort. The patients were divided into two study cohorts: Total Cohort and a Total Hip Arthroplasty (THA) Cohort. The Total Cohort included patients who underwent either THA and revision THA procedures, while the THA Cohort included only patients who underwent a THA.

The data sets were then analyzed to determine total numbers, frequencies, means and standard deviations for key clinical values. Univariate and stepwise forward logistic regression analyses were utilized in order to determine the statistical relationships between days pre-op and our chosen measures of quality: LOS, ICU admission frequency, and major complication frequency. The variable “Days pre-op” refers to how many days prior to the date of surgery the pre-operative evaluation occurred. Major complications included: myocardial infarction, pulmonary embolism, acute renal failure, cerebral vascular accident, hypotension, circulatory failure, and respiratory failure. ASA classification was used as a general surrogate for patients’ health. A two-tailed Fisher’s t-test was used in order to detect statistical significance, while a p-value of < 0.05 was deemed statistically significant. The time intervals were chosen based on the time points at which a pre-surgical clearance may occur pre-operatively. Time intervals of 5-days, 10-days, and 30-days were analyzed, while statistically significant differences in the quality of care mean values were presented. In addition, a power analysis shows that a minimum cohort number of 17 was required for sufficient statistical power—assuming a power of 80% and a statistically significant p-value of < 0.05 for the LOS variable. All comparison cohorts contained more than n=52 samples each, meeting the statistical power threshold. The study examined differences in clinical factors varying days of pre-operative medical evaluation.

## RESULTS

Data was analyzed for the respective patient groups: Total Cohort (n=167) and THA Cohort (n=144). The demographic and clinical data of these study cohorts are listed in Table **1**. The mean clinical values, along with their standard deviations and p-values are presented in Table **2**. The two cohort populations did not differ statistically for any of these clinical values.

There was a correlation between fewer days pre-operative medical evaluation and lower average LOS. These findings are presented in Table **3**. More specifically, patients who obtained their pre-operative evaluation 4 or less days prior to surgery had a 0.58 day (p=0.03) reduction in the LOS in the Total Cohort, and a 0.53 day reduction (p=0.04) in the LOS in the THA Cohort. Additionally, patients who obtained their pre-operative evaluation 6 or less days prior to surgery had a 0.45 day (p=0.02) reduction in the LOS in the Total Cohort, and a 0.41 day reduction (p=0.04) in LOS in the THA Cohort.

Evident in Table **4**, the study found that fewer pre-op days directly correlated with lower ICU admission frequency. This was found to be true for patients who obtained their pre-operative evaluation 4 or fewer days prior to surgery, compared to 5 days or more prior to surgery, as well as 10 days or fewer prior to surgery, compared to 11 days or more prior to surgery in both the study cohorts. Additionally, within the Total Cohort, it was found that patients obtaining their pre-operative evaluation 11 days or less prior to their procedure date had a lower ICU admission frequency compared to those obtaining their pre-operative evaluation 12 days or more prior to their procedure (0.06 vs. 0.15; p=0.04).

As outlined in Table **5**, fewer pre-op days was correlated with lower major complication frequency. Specifically, patients who obtained their pre-operative evaluation 11 or less days prior to surgery had a lower major complication frequency compared to those who obtained their pre-operative evaluations 12 or more days before surgery in the Total Cohort (0.00 vs. 0.03; p<0.05), and in the THA Cohort (0.00 vs. 0.04; p<0.05).

There were no statistically significant differences in age, ASA class, or BMI between the patient populations undergoing pre-operative evaluations 4 days or fewer compared to 5 days or more prior to surgery (Table **6**). This was found in both the Total Cohort as well as the THA Cohort.

The patient population undergoing pre-operative evaluation 11 days or less prior to surgery had, on average, a lower ASA class than those undergoing pre-operative evaluations 12 days or more prior to surgery in the Total Cohort (2.2 vs. 2.4; p=0.04). However, this difference was not significant in the THA Cohort (2.2 vs. 2.4; p=0.07). Neither age nor BMI were significantly different in either cohort or population (Table **7**).

## DISCUSSION

As the rates of total joint arthroplasty procedures in the United States rise, it remains vital to identify processes that may improve quality and care outcomes [16-18]. As reflected in the literature, the effective optimization of patients prior to surgery is a key component to improving perioperative outcomes [19-21]. In fact, pre-operative evaluations can often illuminate previously unknown comorbidities, along with a variety of other potentially risky conditions [22, 23]. Additionally, pre-operative evaluations have been shown to decrease the risk of mortality, total costs of patient care, and a variety of other outcome measures [24-26].

While there are many studies in the medical and orthopedic literature examining the role of pre-operative evaluations, these studies mostly focus on the implementation of different pre-operative interventions and their effects on outcomes [27-29]. While it is certainly important to determine what elements of a pre-operative evaluation may contribute to better outcomes, we hypothesize the importance of examining the timing of pre-operative evaluations and the effect on patient outcomes. However, little investigation has focused on identifying the optimal timing of pre-operative evaluations, especially for THA. We know of no previous studies examining this relationship within the orthopedic literature [15]. Thus, this study attempts to address this literature gap.

The study’s first significant findings were multiple correlations between fewer days pre-op and lower average LOS. This data appears to indicate that scheduling pre-operative assessments closer in time to the procedure date may improve care outcomes by reducing LOS. There may be a number of reasons for this observation, and potentially many confounding factors that can contribute to this difference in the LOS. This may be due to increased patient adherence to physician guidelines or simply due to a difference in the patient population [30].

The second significant finding of the study was a correlation between fewer days pre-op and lower ICU admission frequency. Patients who obtained their pre-operative evaluations closer in time to their procedure date had significantly lower ICU admission frequencies. In fact, no patient undergoing a pre-operative evaluation less than five days prior to surgery was admitted to the ICU. These findings were replicated both in the Total Cohort as well as the THA Cohort, and may indicate that scheduling pre-operative assessments closer in time to the procedure date may reduce the risk of an ICU admission due to optimization measures closer to the surgical intervention.

The third outcome measure used within the study was major complication frequency. The study found that fewer pre-op days correlated with lower major complication frequency. Specifically, patients undergoing pre-operative evaluations 11 days or less prior to surgery exhibited a lower major complication frequency than those who obtained their evaluations 12 days or more prior to their procedure in both the Total Cohort as well as the THA Cohort. In fact, no major complications occurred for any patient within the study that obtained a pre-operative evaluation less than 12 days prior to their procedure. This seems to indicate that scheduling a pre-operative evaluation closer in time to the procedure date may be a preventative measure against major complications. This may be due to the pre-operative evaluation’s ability to gather more accurate data immediately prior to surgery, limiting the window in which new co-morbidities or conditions may arise prior to surgery [31, 32].

While this study found that pre-operative evaluations closer to the procedure date correlated with improved outcomes, as measured by LOS, ICU admission frequency, and major complication frequency, it is important to note that this does not implicate a causal relationship due to the nature of this retrospective dataset. In order to better understand if the significant differences in outcomes found within the study were due to the timing of pre-operative evaluations or other factors, an analysis of the comparative population groups was conducted. This analysis of patient subgroups indicated that there were no statistically significant differences in age, ASA class, or BMI for any of the population groups, except for the ASA class of patients in the Total Cohort’s 11-day pre-op cutoff populations. While there were no other statistically significant differences in age, BMI, or ASA class for any of the other comparative populations, it is important to note that the mean age, BMI, and ASA class were all elevated for each population group above the 11-day pre-op cutoff. This may indicate that patients who received their pre-operative evaluations earlier were simply less healthy, which may account for the significant differences found in outcomes measures. This may be due to a number of reasons, chiefly that physicians may schedule patients perceived to be riskier for earlier consultations pre-operatively in order to better assess surgical risk. This is a potential confounding variable within our study, as well as a limitation to our results. Additionally, it is important to note that an analysis of co-morbidities was not included in this study, as relevant data was not attainable.

While the study identified a number of critical relationships that indicate shorter time periods between pre-operative evaluations and procedure dates may improve perioperative outcomes, there are a number of limitations of the study that must be considered. First, the sample size of the pilot study (n=167) may be too small to draw broad conclusions, although the sample sizes and comparison groups met the threshold for statistical power analysis. Second, the time intervals chosen for subgroup analysis may experience selection bias, as they were chosen based on when pre-surgical clearance might occur pre-operatively. Time intervals of 5-days, 10-days, and 30-days were analyzed, while statistically significant differences in the quality of care mean values were presented. Additionally, the study data was gathered from one institution, and thus may not be generalizable to other institutions. As a retrospective study of correlations, no causal relationship can be concluded. Thus, although the study found that shorter times between pre-operative evaluations and procedures correlated with improved perioperative outcomes, the study may not conclude that these improved outcomes were due to the timing of pre-operative evaluations alone. Further, although the results discussed within the study exhibited statistical significance, the differences observed in the LOS, ICU admission frequency, and major complication frequency may hold less clinical significance in a practice setting than statistical significance. In addition, new issues found 4 days prior to surgery may be difficult or even impossible to address fully in a clinical setting. These differences in outcomes may be due to a variety of factors, such as differences in the health of the compared populations, differences in treatment, and many other confounding factors [33, 34]. Certainly, this is an area which needs further research.

## CONCLUSION

In conclusion, as the THA procedure volume continues to grow, it becomes more important to identify potential processes that may improve surgical outcomes. A vital aspect of any medical procedure is the reduction of risk through pre-operative evaluations. This study sought to examine whether or not the timing of pre-operative evaluations may contribute to perioperative outcomes, a novel topic absent from the present literature. While this study finds that pre-operative evaluations closer to the procedure date are correlated with improved outcomes, additional studies must be performed in order to validate this conclusion.

## Figures and Tables

**Table 1:** Demographic and clinical data of study cohorts.

**Variable**	**Total Cohort** N=167 (%)	**THA Cohort** N=144 (%)
**Age**		
≤50	35 (21.0%)	31 (21.5%)
50-60	51(30.5%)	42 (29.2%)
60-70	50 (29.9%)	41 (28.5%)
70-80	21 (12.6%)	20 (13.9%)
≥80	10 (6.0%)	10 (6.9%)
**Gender**		
Male	80 (47.9%)	66 (45.8%)
Female	87 (52.1%)	78 (54.2%)
**Procedure**		
THA	144 (86.2%)	144 (100.0%)
Revision THA	23 (13.8%)	0 (0.0%)
**ASA Score**		
1	6 (3.6%)	6 (4.2%)
2	94 (56.3%)	81 (56.3%)
3	67 (40.1%)	57 (39.6%)
**BMI**		
<25	44 (26.3%)	39 (27.1%)
25-29.9	49 (29.3%)	42 (29.2%)
30-34.9	50 (29.9%)	44 (30.6%)
35-39.9	12 (7.2%)	11 (7.6%)
≥40	12 (7.2%)	8 (5.6%)
**Days Pre-Op**		
≤7	31 (18.6%)	28 (19.4%)
8-14	49 (29.3%)	45 (31.3%)
15-21	34 (20.4%)	30 (20.8%)
≥22	53 (31.7%)	41 (28.5%)
**Pre-Op Type**		
Internal Medicine	99 (59.3%)	99 (68.8%)
Other	68 (40.7%)	45 (31.3%)
**Length of Stay**		
≤3	9 (5.4%)	7 (4.9%)
4	112 (67.1%)	99 (68.8%)
5	28 (16.8%)	25 (17.4%)
6	8 (4.8%)	6 (4.2%)
≥7	10 (6.0%)	7 (4.9%)
**ICU Admission**		
Yes	20 (12.0%)	12 (8.3%)
No	147 (88.0%)	132 (91.7%)
**Major Complication**		
Yes	4 (2.4%)	4 (2.8%)
No	163 (97.6%)	140 (97.2%)

**Table 2:** Mean clinical values, standard deviations, and P-values of study cohorts.

**Variable**	**Total Cohort**	**THA Cohort**	**P-Value**
Mean Age ± s.d.	57.9 ± 14.2	58.0 ± 15.0	0.93
Mean ASA Class ± s.d.	2.4 ± 0.6	2.4 ± 0.6	0.86
Mean BMI ± s.d.	29.4 ± 6.5	29.1 ± 6.2	0.70
Mean Days Pre-Op ± s.d.	18.9 ± 14.3	18.3 ± 14.3	0.86
Mean Length of Stay ± s.d.	4.4 ± 1.5	4.4 ± 1.4	0.78

**Table 3:** Significant differences in mean length of stay by days pre-op.

**Total Cohort**			**THA Cohort**		
**Days Pre-Op**	**Mean LOS**	**P-Value**	**Days Pre-Op**	**Mean LOS**	**P-Value**
≤4 vs. ≥5	3.91 vs. 4.49	0.03	≤4 vs. ≥5	3.91 vs. 4.44	0.04
≤6 vs. ≥7	4.05 vs. 4.50	0.02	≤6 vs. ≥7	4.05 vs. 4.46	0.04

**Table 4:** Significant differences in ICU admission frequency by days pre-op.

**Total Cohort**			**THA Cohort**		
**Days Pre-Op**	**ICU Admission Frequency**	**P-Value**	**Days Pre-Op**	**ICU Admission Frequency**	**P-Value**
≤4 vs. ≥5*	0.00 vs. 0.13	<0.01	≤4 vs. ≥5*	0.00 vs. 0.09	<0.01
≤10 vs. ≥11	0.04 vs. 0.15	0.01	≤10 vs. ≥11	0.02 vs. 0.11	0.03
≤11 vs. ≥12	0.06 vs. 0.15	0.04			

*All procedures with less than 5 pre-op days were statistically significant in comparison, but not shown.

**Table 5:** Significant differences in major complication frequency by days pre-op.

**Total Cohort**			**THA Cohort**		
**Days Pre-Op**	**Major Complication Frequency**	**P-Value**	**Days Pre-Op**	**Major Complication Frequency**	**P-Value**
≤11 vs. ≥12*	0.00 vs. 0.03	<0.05	≤11 vs. ≥12*	0.00 vs. 0.04	<0.05

*All procedures with less than 12 pre-op days were statistically significant in comparison, but not shown.

**Table 6:** Differences in mean clinical factors at 4-day pre-op cutoff for total and THA cohort.

Variable	**Total Cohort**		**P-Value**	**THA Cohort**		**P-Value**
	≤4 Days	≥5 Days		≤4 Days	≥5 Days	
Mean Age ± s.d.	51.1 ± 17.4	58.4 ± 13.9	0.20	51.1 ± 17.4	58.6 ± 14.7	0.19
Mean ASA Class ± s.d.	2.3 ± 0.5	2.4 ± 0.6	0.52	2.3 ± 0.5	2.4 ± 0.6	0.55
Mean BMI ± s.d.	27.8 ± 7.8	29.5 ± 6.4	0.48	27.8 ± 7.8	29.2 ± 6.0	0.57

**Table 7:** Differences in mean clinical factors at 11-day pre-op cutoff for total and THA cohort.

**Variable**	**Total Cohort**		**P-Value**	**THA Cohort**		**P-Value**
	≤11 Days	≥12 Days		≤11 Days	≥12 Days	
Mean Age ± s.d.	55.5 ± 14.8	59.0 ± 13.9	0.14	55.5 ± 14.0	59.4 ± 14.8	0.15
Mean ASA Class ± s.d.	2.2 ± 0.5	2.4 ± 0.6	0.04	2.2 ± 0.5	2.4 ± 0.6	0.07
Mean BMI ± s.d.	28.4 ± 6.6	29.9 ± 0.2	0.17	28.0 ± 6.5	29.7 ± 5.9	0.14
